# BACH1-mediated transcriptional repression of pro-angiogenic factors drives angiogenic impairment in hypertension

**DOI:** 10.3389/fcvm.2026.1769747

**Published:** 2026-02-11

**Authors:** Datian Gao, Zhiwen Wu, Zhiyin Zhou, Jianwen Liang

**Affiliations:** Department of Cardiology, The Eighth Affiliated Hospital, Sun Yat-sen University, Shenzhen, Guangdong, China

**Keywords:** angiogenesis, BACH1, endothelial, hypertension, transcription

## Abstract

**Background:**

Impaired angiogenesis is a well-recognized pathophysiological feature of hypertension, yet the molecular mechanisms linking elevated blood pressure to angiogenic impairment remain incompletely understood. Endothelial transcriptional regulation may play a critical role in this process.

**Methods:**

Angiogenesis was assessed in Angiotensin II (AngII)-induced hypertensive mice and endothelial cells using *in vivo* and *in vitro* approaches. BACH1 expression and regulation were examined following AngII stimulation. Transcriptional repression by BACH1 was investigated at pro-angiogenic gene promoters. Circulating angiogenic factors were measured in hypertensive patients and analyzed in relation to blood pressure. Endothelial-enriched BACH1 knockdown was performed *in vivo* to evaluate its effects on angiogenesis and blood pressure.

**Results:**

Angiogenesis was significantly impaired in AngII-induced hypertensive mice and endothelial cells, accompanied by marked upregulation of BACH1. Mechanistically, BACH1 acted as a direct transcriptional repressor by binding to the promoters of key pro-angiogenic factors, including FGF1, VEGFA, ANGPT1 and AGGF1, thereby suppressing their expression. Consistently, circulating levels of these factors were reduced in hypertensive patients and negatively correlated with blood pressure. Importantly, endothelial-enriched BACH1 knockdown restored retinal angiogenesis and significantly attenuated hypertension development in AngII-treated mice.

**Conclusion:**

These findings identify BACH1 as a critical transcriptional regulator linking hypertension to impaired angiogenesis and suggest that targeting endothelial BACH1 may represent a potential therapeutic strategy for hypertension.

## 1 Introduction

Hypertension is a leading global health concern, affecting over 1.4 billion people and representing a primary risk factor for cardiovascular morbidity and mortality (1, 2). The increasing prevalence of hypertension, driven by population aging and lifestyle factors, imposes a substantial and growing burden on global healthcare systems and economies (3). Despite the availability of anti-hypertensive therapies, the rates of awareness, treatment, and control remain suboptimal, underscoring the need to identify novel therapeutic targets for its prevention (1, 4).

Angiogenesis refers to the process through which new blood vessels branch out from pre-existing vessels via sprouting (5, 6). This complex process is regulated by various factors, including vascular endothelial growth factor (VEGF), fibroblast growth factor (FGF), angiopoietins, platelet-derived growth factor (PDGF) and Notch signaling (7–10). Recent studies have also highlighted the critical role of angiogenic factor with G patch and FHA domains 1 (AGGF1) in regulating retinal angiogenesis (11). Insufficient angiogenesis is an important pathophysiological characteristic of hypertension. Several studies, including our previous work, have demonstrated impaired angiogenesis in hypertensive patients and Angiotensin II (AngII)-induced hypertensive mouse models (12, 13). However, the precise molecular mechanisms that link hypertension to deficient angiogenesis remain to be further investigated.

BTB and CNC homology 1 (BACH1) is an important member of the Cap “n” Collar and basic region leucine zipper (CNC-bZIP) family. As a key transcriptional regulator, it participates in the modulation of diverse biological processes, including heme homeostasis, oxidative stress and cell-cycle progression (14–16). BACH1 is closely associated with cardiovascular diseases. Studies have demonstrated that BACH1 knockout significantly attenuates atherosclerotic lesions and ameliorates endothelial inflammation (17). Furthermore, small-molecule compounds targeting BACH1 have been shown to improve vascular regeneration in ischemic vascular diseases (18). However, the role of BACH1 in hypertension and its relationship with impaired angiogenesis in this condition remain to be fully explored.

In this study, we establish a previously unrecognized role for BACH1 as a key mediator of hypertension-induced angiogenesis deficiency. We observed that BACH1 expression is significantly upregulated in endothelial cells following stimulation with AngII, a central mediator of hypertension. Critically, inhibition of BACH1 led to a marked increase in the expression of key pro-angiogenic factors, namely FGF1, VEGFA, ANGPT1, and AGGF1. Consistent with these findings, we identified a significant negative correlation between the circulating levels of these pro-angiogenic factors and blood pressure. To validate these findings *in vivo*, specific knockdown of endothelial BACH1 markedly improved impaired angiogenesis in AngII-induced hypertensive mice. This study is the first to demonstrate a pivotal role for BACH1 in linking hypertension to angiogenesis deficiency. Our findings reveal a novel mechanistic pathway and suggest that targeting BACH1 may represent a promising and innovative therapeutic strategy to restore vascular function and improve clinical outcomes in hypertensive patients.

## 2 Methods

### 2.1 Human samples

This study was conducted in accordance with protocols approved by the ethics committee of The Eighth Affiliated Hospital of Sun Yat-sen University (Shenzhen, China). Following informed consent, serum samples were procured from 32 patients diagnosed with hypertension and 30 normotensive, healthy volunteers. The collected serum was utilized to quantify the concentrations of FGF1, VEGFA, ANGPT1, and AGGF1 using commercial ELISA kits. A comprehensive summary of the participants' clinical characteristics is detailed in Supplementary Table 1.

### 2.2 Animal models

All animal experiments were performed under the ethical guidelines and approval of the Animal Care and Use Committees at The Eighth Affiliated Hospital of Sun Yat-sen University. To induce hypertension, C57BL/6J mice were subjected to a continuous 4-week subcutaneous infusion of Angiotensin II (AngII, Sigma-Aldrich) at a rate of 0.8 mg/kg/day, delivered via surgically implanted osmotic mini-pumps. A control group received saline infusions under identical conditions. Systolic blood pressure was assessed throughout the study using a Softron BP-2010A noninvasive tail-cuff system.

### 2.3 Anesthesia and euthanasia

All animal experiments were conducted in accordance with institutional guidelines and approved protocols for the care and use of laboratory animals.

Animals were anesthetized using isoflurane inhalation anesthesia. Anesthesia was induced with 4% isoflurane and maintained at 1%–2% isoflurane delivered in oxygen using an anesthesia machine. Adequate depth of anesthesia was confirmed by the absence of pedal withdrawal reflex prior to experimental procedures.

At the end of the experiment, animals were humanely euthanized by an overdose of anesthetic, followed by confirmation of death through cessation of respiration and heartbeat. All efforts were made to minimize animal suffering.

### 2.4 Endothelium-Targeted BACH1 silencing *in vivo*

For endothelial-specific gene knockdown, 8-week-old male C57BL/6J mice received a single tail vein injection of recombinant AAV9 vectors (5 × 10^11^ viral genomes/mouse) encoding either a BACH1-targeting siRNA (5'- GGAUCUACUACCAGGAGUATT -3') or a non-targeting scrambled control. One week after vector administration to allow for transduction, hypertension was induced with a 28-day AngII infusion (0.8 mg/kg/day). Blood pressure was assessed on a weekly basis, and at the study's conclusion, aortic tissues were harvested for subsequent morphological evaluation.

### 2.5 Cell culture and treatment

Human umbilical vein endothelial cells (HUVECs) were cultured in endothelial-specific growth medium (ScienCell) containing 10% fetal bovine serum (FBS, Gibco) and 1% penicillin/streptomycin (Gibco). For stimulation experiments, HUVECs were treated with 10^−6^ M AngII (AmBeed) for 48 h.

Human 293 T cells were cultivated in DMEM containing 10% FBS and 1% penicillin-streptomycin. Both cell lines were maintained in a humidified incubator at 37 °C with 5% CO₂.

### 2.6 Aortic pulse wave velocity measurement *in vivo*

To assess arterial stiffness, aortic pulse wave velocity (aPWV) was measured in mice anesthetized with 2% isoflurane. With continuous ECG monitoring, Doppler probes were placed consecutively over the aortic arch and the abdominal aorta. The pulse transit time was derived from the interval between the ECG R-wave and the foot of the Doppler signal at each location. The aPWV was then calculated as the ratio of the distance between the probes to the differential transit time.

### 2.7 Aortic vasorelaxation assay *in vitro*

Aortic rings (2 mm) were excised from euthanized mice and mounted in a multi-wire myograph system (DMT620M) containing Krebs solution aerated with 5% CO₂ at 37 °C for isometric tension studies. After equilibration, the functional integrity of the rings was confirmed by their contractile response to 1 μM phenylephrine (PE) and subsequent endothelial-dependent relaxation with 30 μM acetylcholine (ACh). Cumulative concentration-response curves to the vasodilator sodium nitroprusside were subsequently generated. Vasorelaxation was expressed as the percentage reversal of the PE-induced pre-constriction.

### 2.8 Isolation and culture of primary mouse aortic endothelial cells (MAECs)

Primary aortic endothelial cells (MAECs) were obtained from C57BL/6J mouse aortas using an explant culture technique. Aortic segments were placed lumen-side down on dishes coated with growth factor-reduced Matrigel (Corning) and cultured in ECM medium (Cellvita, 1010, Shanghai Medicell Biotechnology Co.,Ltd.) supplemented with growth factors and 10% FBS. After 4 days, endothelial cells that had migrated from the explants were expanded. The endothelial phenotype of the cultured MAECs was confirmed by assessing CD31 expression via flow cytometry.

### 2.9 Enzyme linked immunosorbent assay (ELISA)

Serum FGF1, VEGFA, ANGPT1 and AGGF1 concentrations were assessed by individual ELISA kits (R&D Systems, Abingdon, UK) following the manufacturer's instructions. Optical densities were recorded in a Multiskan GO spectrophotometer (Thermo Scientific, Waltham, USA) at 450 nm with a wavelength correction at 540 nm.

### 2.10 Small interfering RNA (siRNA) transfection

For *in vitro* gene silencing, siRNAs were prepared at a final concentration of 50 nmol/L. Transfection into cells was achieved using NanoTrans™ Transfection Reagent 3000 (CYTOCH), following the provided guidelines. Cells were collected at 48 h after transfection. The specific siRNA sequences are listed in Supplementary Table S2.

### 2.11 Endothelial cell tube formation

The angiogenic potential of HUVECs was assessed using a tube formation assay. Wells of a 96-well plate were coated with 80 μL of growth factor-reduced Matrigel and allowed to polymerize at 37 °C. HUVECs (1.5 × 10⁴ cells/well) were then seeded onto the Matrigel surface. After a 6-hour incubation period, the formation of capillary-like networks was documented with phase-contrast microscopy, and the total tubule length was quantified using the angiogenesis analysis tools in ImageJ.

### 2.12 RNA extraction and quantitative real-time PCR (RT-qPCR)

Total RNA from cultured cells or tissue samples was isolated with TRIzol reagent (Invitrogen). Gene expression levels were quantified by RT-qPCR following manufacturer's protocols. A detailed list of primer sequences used for amplification is provided in Supplementary Table S3.

### 2.13 Western blot analysis

Protein lysates from cells and tissues were prepared using RIPA buffer (NCM, WB3100, China) containing a protease/phosphatase inhibitor cocktail and PMSF. Tissue samples were mechanically homogenized, while cell lysates were prepared by scraping. Protein concentration was determined using the BCA assay (Pierce™). Equal amounts of protein were resolved by SDS-PAGE, transferred to membranes, and probed with specific primary antibodies. Information regarding the antibodies is available in Supplementary Table S4.

### 2.14 Luciferase assay

To investigate transcriptional regulation, 293 T cells were co-transfected with a pGL4.10 vector containing the FGF1, VEGFA, ANGPT1 and AGGF1 promoter, a pcDNA3.1-BACH1 expression vector, and a pRL-TK Renilla luciferase control vector using Lipofectamine 3000 (Invitrogen). At 48 h post-transfection, luciferase activity was measured using the Dual-Luciferase Reporter Assay System (Promega). Firefly luciferase signals were normalized against the Renilla luciferase activity to correct for transfection efficiency.

### 2.15 Chromatin immunoprecipitation (ChIP)-qPCR analysis

ChIP assays were performed using the Pierce Magnetic ChIP Kit (Thermo Scientific) according to the supplier's protocol. Briefly, cells were cross-linked with formaldehyde, and chromatin was sonicated to an average size of ∼300 bp. The sheared chromatin was then subjected to immunoprecipitation overnight with antibodies against BACH1 or a non-specific IgG control. Following DNA elution and purification, enrichment of the FGF1, VEGFA, ANGPT1 and AGGF1 promoter region were quantified using SYBR Green-based qPCR. The primer sequences are detailed in Supplementary Table S5.

### 2.16 Immunofluorescence and confocal microscopy

Paraffin-embedded mouse aortic sections were processed for immunofluorescence analysis. Following antigen retrieval and permeabilization, sections were blocked and then probed overnight at 4 °C with primary antibodies targeting BACH1. Staining was visualized using corresponding Alexa Fluor-conjugated secondary antibodies and DAPI for nuclear counterstaining. Images were acquired on a Zeiss LSM 880 confocal microscope, and fluorescence intensity analysis was conducted with ImageJ software.

### 2.17 Statistical analysis

Results are expressed as the mean ± standard deviation (SD) for continuous variables and as frequencies (%) for categorical data. For two-group comparisons, a two-tailed Student's *t*-test was utilized. For multi-group comparisons, one-way ANOVA followed by Tukey's *post hoc* tests was employed. Correlations between variables were evaluated using Pearson's coefficient. A *P*-value less than 0.05 was considered statistically significant. All statistical computations were performed using GraphPad Prism 9.0.

## 3 Results

### 3.1 The capacity for angiogenesis is significantly reduced in hypertension

**Figure 1:**
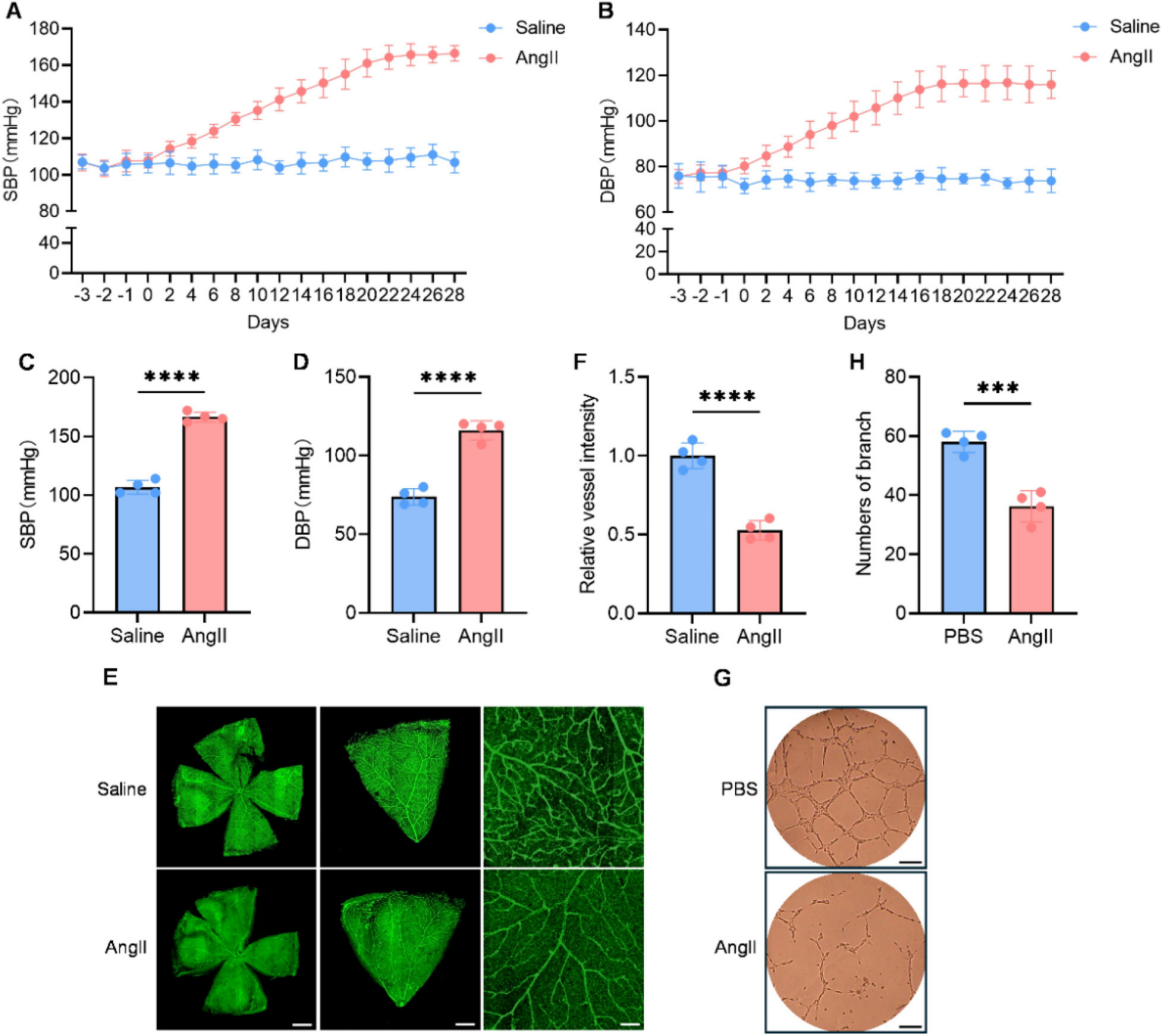
The capacity for angiogenesis is significantly reduced in hypertension. **(A**–**D)** The systolic blood pressure (SBP; A and C) and diastolic blood pressure (DBP; B and D) level of hypertensive mice, control mice were detected by a tail-cuff plethysmography system (*n* = 4). **(E,F)** Representative images of retinal vascular visualized by isolectin-B4 staining. Scale bars: 500 μm (row1), 100 μm (row2), 50 μm (row3), (*n* = 4). **(G,H)** Tube formation assay of endothelial cells treated with PBS or AngII (*n* = 4). Data were shown as mean ± s.d. ***P < 0.001, ****P < 0.0001. Statistical analysis was performed by two-tailed Student's *t*-test (**C,D,F** and H).

To investigate the impact of hypertension on angiogenesis, we established a hypertensive mouse model by continuous subcutaneous infusion of Angiotensin II (AngII, 0.8 mg/kg/day) via osmotic pumps for 4 weeks. Non-invasive monitoring confirmed a significant elevation in both systolic and diastolic blood pressure in the AngII-treated group relative to the saline-infused mice (Figures 1A–D). Furthermore, vascular function assessments revealed that AngII administration increased aortic pulse wave velocity (aPWV) and impaired vasorelaxation, indicative of significant vascular damage (Supplementary Figures S1A,B). To directly assess angiogenesis *in vivo*, we performed isolectin B4 (IB4) staining of retinal flat mounts. The results demonstrated a marked reduction in retinal vascular density in hypertensive mice (Figures 1E,F). These *in vivo* findings were corroborated by *in vitro* experiments, where AngII treatment significantly suppressed the tube formation capacity of endothelial cells (Figures 1G,H). Collectively, these data provided clear evidence from both *in vivo* and *in vitro* models that hypertension is characterized by deficient angiogenesis.

### 3.2 BACH1 emerges as a critical regulator of hypertension-associated angiogenic impairment

**Figure 2:**
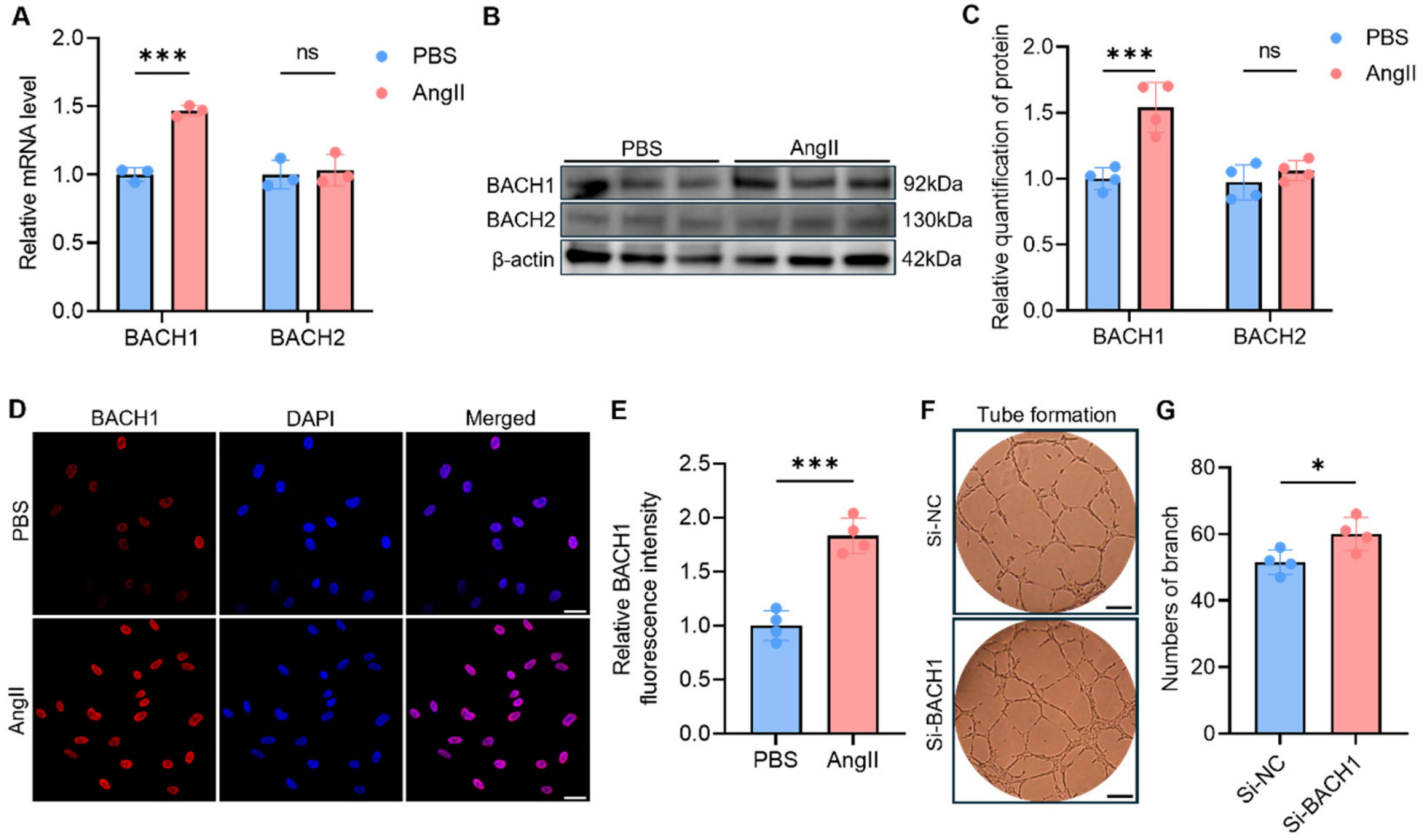
BACH1 emerges as a critical regulator of hypertension-associated angiogenic impairment. **(A)** mRNA levels of BACH1 and BACH2 were measured using quantitative PCR in HUVECs following AngII or PBS stimulation (*n* = 3). **(B,C)** Western blotting detection of BACH1 and BACH2 protein expression in HUVECs following different treatment (*n* = 4). **(D,E)** Immunofluorescence of the BACH1 (red) and DAPI-stained nuclei (blue) in differential treatment (*n* = 4). Scale ba*r* = 20 μm. **(F,G)** Tube formation assay of endothelial cells treated with Si-NC or Si-BACH1 (*n* = 4). Data were shown as mean ± s.d. *P < 0.05, **P < 0.01, ***P < 0.001. Statistical analysis was performed by one-way ANOVA with Tukey's *post hoc* test **(A)**, by two-tailed Student's *t*-test (C, E and G).

While the BACH family of transcription factors is known to play important roles in complex biological processes, including ischemic cardiovascular diseases, its potential involvement in hypertension has not been investigated. Therefore, we sought to determine whether BACH family members contribute to the pathogenesis of hypertension. Following treatment with AngII, we observed a specific and significant upregulation of BACH1 mRNA, whereas BACH2 transcript levels were unaffected in HUVECs (Figure 2A). This selective induction was further validated at the protein level, as demonstrated by western blotting assays (Figures 2B,C). Furthermore, immunofluorescence assays demonstrated that BACH1 is localized within the nucleus, and its intensity was markedly enhanced by AngII stimulation (Figures 2D,E).

Having established that BACH1 is upregulated by AngII stimulus, we next investigated its functional role in angiogenesis. We used small interfering RNA to knockdown BACH1 (Si-BACH1) in HUVECs, the efficiency was validated by qPCR and Western Blotting (Supplementary Figures S2A–C). Using an *in vitro* tube formation assay, we found that the genetic silence of BACH1 was sufficient to rescue endothelial cell angiogenic potential, leading to enhanced network formation (Figures 2F,G). These results identified BACH1 as a key factor upregulated in hypertension that suppresses angiogenesis, highlighting its potential as a therapeutic target for improving angiogenesis in hypertension.

### 3.3 BACH1 directly represses the transcription of key pro-angiogenic factors *in vitro*

**Figure 3:**
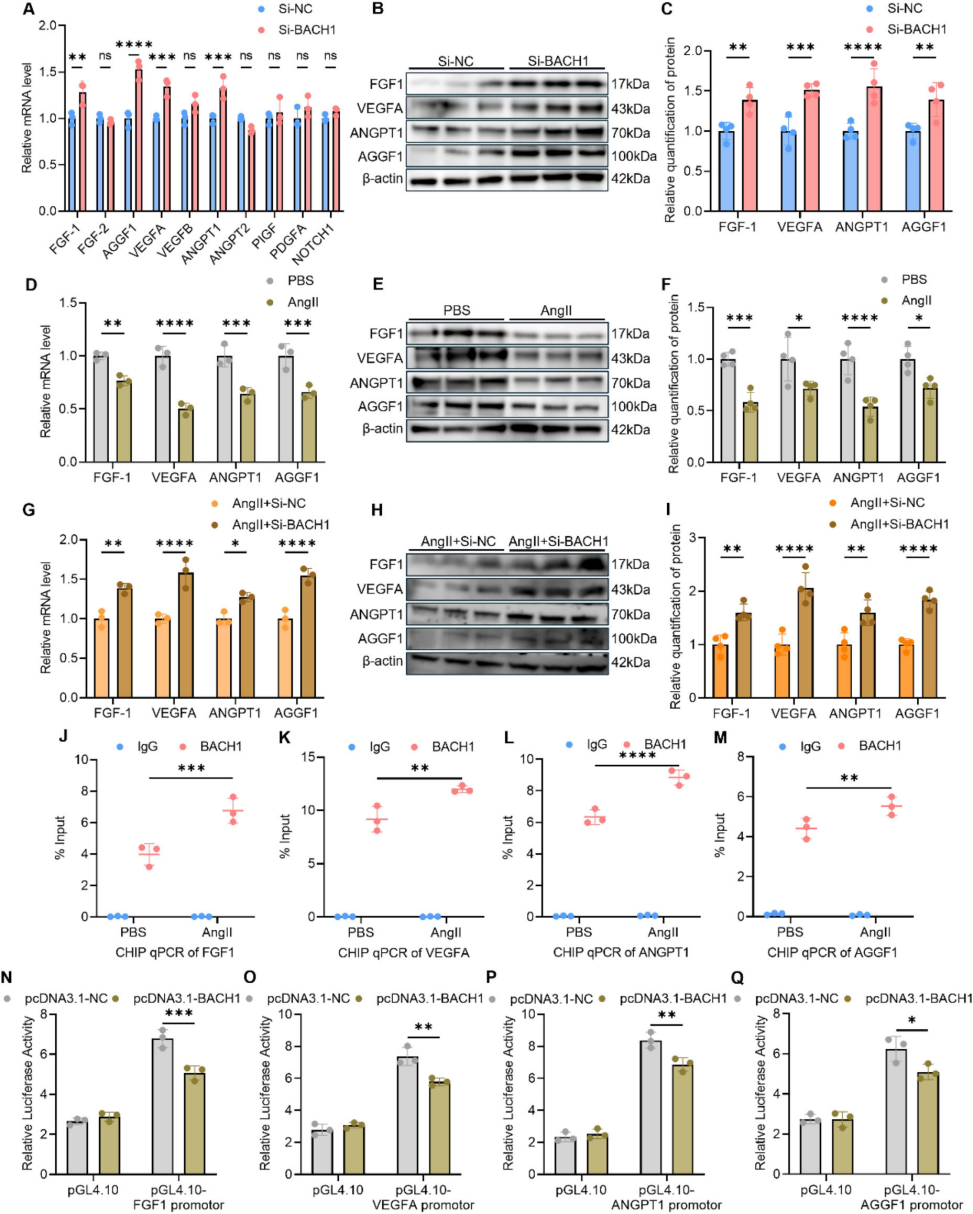
BACH1 directly represses the transcription of key pro-angiogenic factors *in vitro*. **(A)** mRNA levels of series pro-angiogenic factors were measured using quantitative PCR in HUVECs following Si-NC or Si-BACH1 treatment (*n* = 3). **(B,C)** Representative western blot gels of FGF1, VEGFA, ANGPT1 and AGGF1 in Si-NC or Si-BACH1 treated HUVECs (*n* = 4). **(D)** mRNA levels of FGF1, VEGFA, ANGPT1 and AGGF1 were measured in HUVECs following different treatment (*n* = 3). **(E,F)** Protein levels of FGF1, VEGFA, ANGPT1 and AGGF1 in HUVECs treated with PBS or AngII (*n* = 4). **(G)** Transcriptional changes of multiple pro-angiogenic genes in HUVECs subjected to different treatments were analyzed by quantitative PCR (*n* = 3). **(H,I)** Corresponding alterations in FGF1, VEGFA, ANGPT1 and AGGF1 protein expression in HUVECs were determined by western blot analysis (*n* = 4). **(J–M)** Using antibody against BACH1 or IgG, CHIP-qPCR analysis for binding status at the FGF1, VEGFA, ANGPT1 and AGGF1 promotor of PBS or AngII-treated cells (*n* = 3). **(N–Q)** 293 T cells transfected with different plasmids were measured by luciferase assay to assess the interaction between BACH1 and series pro-angiogenic factors (*n* = 3). Data were shown as mean ± s.d. *P < 0.05, **P < 0.01, ***P < 0.001, ****P < 0.0001. Statistical analysis was performed by one-way ANOVA with Tukey's *post hoc* test (A, C-D, F-G, I and J-Q).

Given that angiogenesis is regulated by a complex network of growth factors, we sought to identify the specific downstream targets through which BACH1 exerts its anti-angiogenic effects. Therefore, we performed series of key angiogenic regulators in endothelial cells following BACH1 inhibition. RT-qPCR analysis revealed that knockdown of BACH1 significantly upregulated the mRNA levels of Fibroblast Growth Factor 1 (FGF1), Vascular Endothelial Growth Factor A (VEGFA), Angiopoietin 1 (ANGPT1) and Angiogenic Factor with G-patch and FHA domains 1 (AGGF1). In contrast, the expression of other related factors, including FGF2, VEGFB, ANGPT2, PIGF, PDGFA and NOTCH1, remained unchanged (Figure 3A). This specific response was similarly found at the protein level using western blotting analysis (Figures 3B,C). These findings suggest that BACH1 negatively regulates angiogenesis primarily by suppressing the expression of FGF1, VEGFA, ANGPT1 and AGGF1. To validate the relevance of this axis in hypertensive condition, we treated HUVECs with AngII. As hypothesized, AngII stimulation induced a significant downregulation of FGF1, VEGFA, ANGPT1 and AGGF1 at both mRNA and protein levels (Figures 3D–F), furthermore, inhibition of BACH1 improves levels of FGF1, VEGFA, ANGPT1 and AGGF1 (Figures 3G–I).

Previous studies have established BACH1 as a key transcriptional regulator. We therefore questioned whether BACH1 directly inhibits the expression of these pro-angiogenic factors by binding on their regulatory regions. We performed Chromatin Immunoprecipitation followed by qPCR (ChIP-qPCR) in endothelial cells. The results demonstrated a significantly enhanced recruitment of BACH1 to the promoter regions of *FGF1*, *VEGFA*, *ANGPT1* and *AGGF1* in AngII-treated cells compared to the PBS control group (Figures 3J–M). To confirm this repressive interaction, we conducted dual-luciferase reporter assays, which showed that BACH1 overexpression inhibited the promoter activity of all four angiogenic factors (Figures 3N–Q).

Collectively, these mechanistic data provided evidence that BACH1 acts as a direct transcriptional repressor of *FGF1*, *VEGFA*, *ANGPT1* and *AGGF1*. Under hypertensive conditions, elevated BACH1 levels lead to increased binding at these gene promoters, thereby suppressing their expression and culminating in the inhibition of angiogenesis.

### 3.4 Pro-angiogenic factors negatively correlated with blood pressure *in vivo*

**Figure 4:**
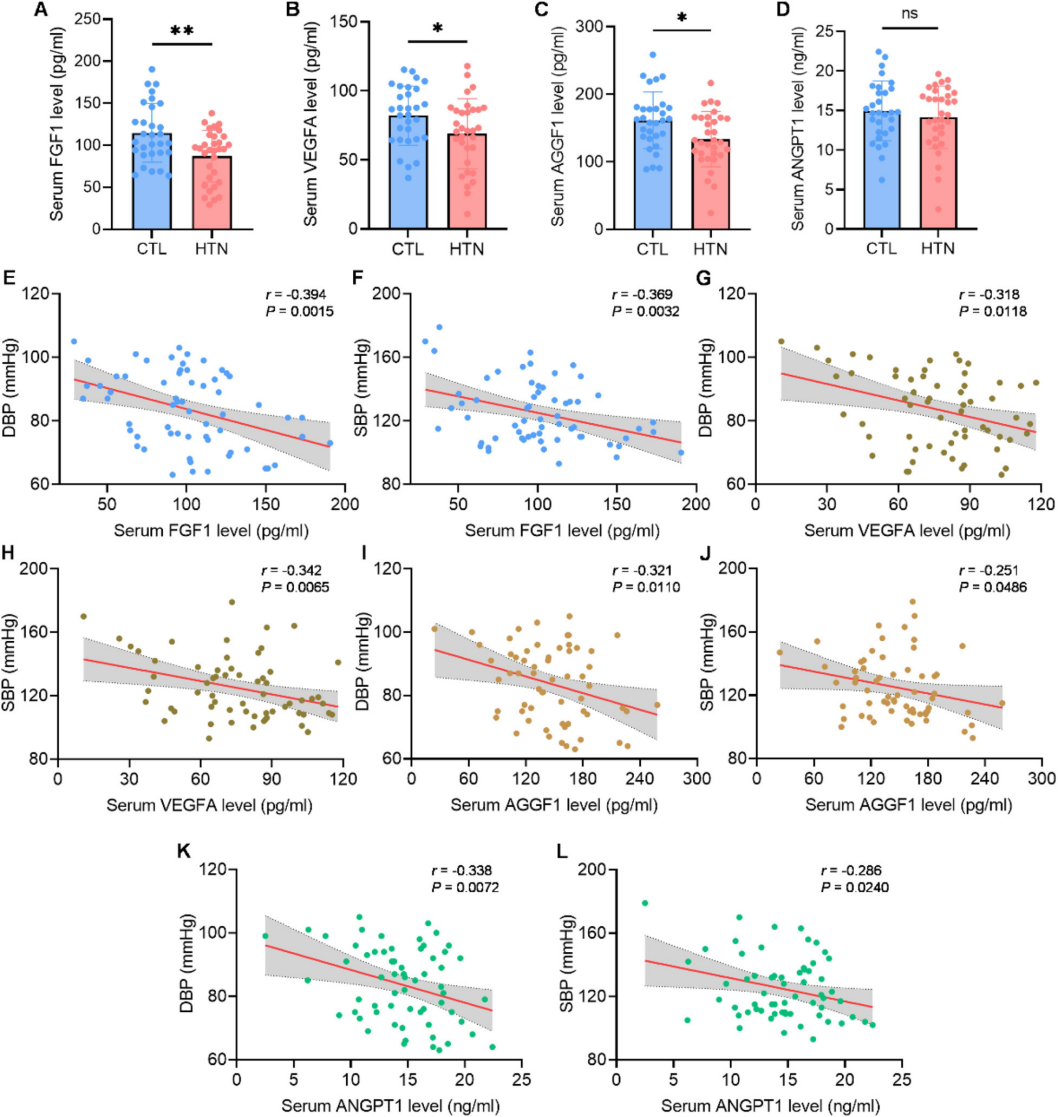
Pro-angiogenic factors negatively correlated with blood pressure *in vivo*. **(A–D)** Quantification of serum FGF1 **(A)**, VEGFA **(B)**, AGGF1 **(C)** and ANGPT1 **(D)** levels via ELISA assays in healthy controls (*n* = 30) and hypertensive patients (*n* = 32). **(E–L)** Pearson's correlation analyses assessing the relationship between serum concentrations of FGF1 **(E,F)**, VEGFA **(G,H)**, AGGF1 **(I,J)**, and ANGPT1 **(K,L)** and corresponding DBP or SBP. Data were shown as mean ± s.d. *P < 0.05, **P < 0.01. Statistical analysis was performed by two-tailed Student's t-test **(A–D)**. .

To clarify the clinical relevance of FGF1, VEGFA, ANGPT1 and AGGF1 in hypertension, we enrolled 32 patients diagnosed with hypertension according to WHO criteria and 30 age-matched normotensive volunteers. Peripheral blood samples were collected, and the serum concentrations of the four angiogenic factors were quantified by ELISA. Consistent with our experimental data, the results revealed that circulating levels of FGF1, VEGFA and AGGF1 were significantly lower in hypertensive patients compared to healthy controls (Figures 4A–C). Although the concentration of ANGPT1 also showed a downward trend in the hypertensive group, this difference did not reach statistical significance (Figure 4D).

To further explore the relationship between these factors and hypertension, we performed a correlation analysis. A significant inverse correlation was observed between the circulating levels of all four factors—FGF1, VEGFA, ANGPT1 and AGGF1—and both systolic and diastolic blood pressure (Figures 4E–L). Notably, despite the lack of a significant difference in its mean concentration between the two groups, ANGPT1 levels still negatively correlated with blood pressure, consistent with the other factors.

Collectively, these clinical data demonstrated that in human, higher blood pressure is associated with lower circulating levels of key pro-angiogenic factors. This suggests that the FGF1, VEGFA, ANGPT1 and AGGF1 axis is dysregulated in hypertension and that interventions aimed at restoring the levels of these factors could represent a viable strategy to improve angiogenic function.

### 3.5 Endothelial-enriched knockdown of BACH1 improved angiogenesis *in vivo*

To definitively establish the regulatory role of BACH1 on pro-angiogenic factors *in vivo*, we employed an adeno-associated virus (AAV9) vector to achieve endothelium-enriched-knockdown of BACH1 (EC-KD) in C57BL/6J mice. Following successful vector delivery, these mice were subjected to AngII infusion to induce hypertensive models.

**Figure 5:**
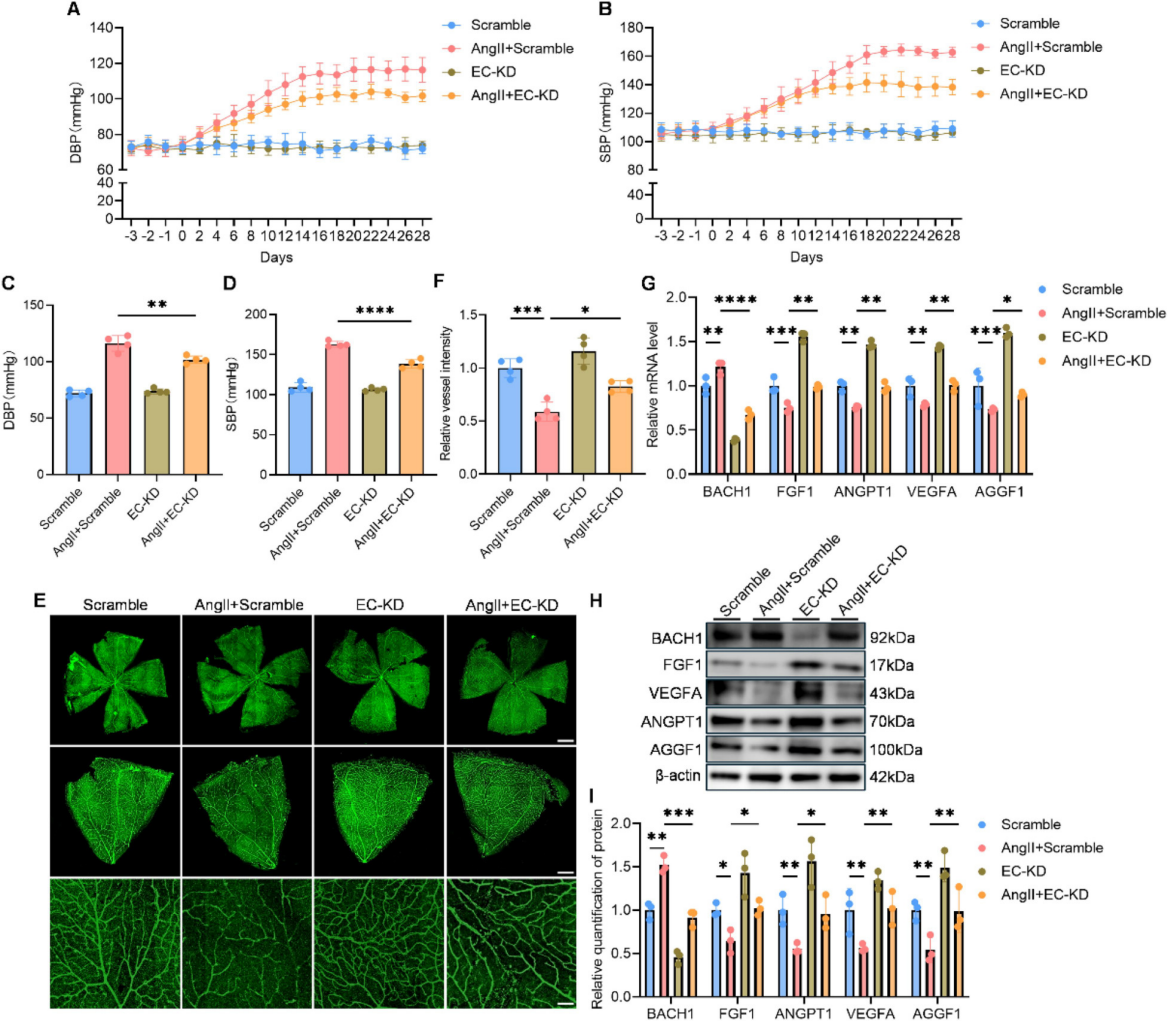
Endothelial-enriched knockdown of BACH1 improved angiogenesis *in vivo*. **(A,B)** Analysis of DBP **(A)** and systolic **(B)** blood pressure, measured by a non-invasive tail-cuff system (*n* = 4). **(C,D)** Endpoint quantification of DBP **(C)** and SBP **(D)** at 28 days (*n* = 4). **(E)** Representative images of retinal vasculature visualized by IB4 staining. Scale bars: 500 μm (row1), 100 μm (row2), 50 μm (row3). **(F)** Quantification of relative vessel intensity (*n* = 4). **(G)** mRNA levels of BACH1, FGF1, ANGPT1, VEGFA and AGGF1 in MAECs isolated from aortas of differentially treated mice (*n* = 3). **(H,I)** Representative western blot gels of BACH1, FGF1, VEGFA, ANGPT1 and AGGF1 in MAECs (*n* = 3). Data were shown as mean ± s.d. *P < 0.05, **P < 0.01, ***P < 0.001, ****P < 0.0001. Statistical analysis was performed by one-way ANOVA with Tukey's *post hoc* test (C, D, F, G and I).

Remarkably, the selective depletion of endothelial BACH1 caused protection against the development of hypertension. Compared to hypertensive mice, endothelial BACH1 knockdown exhibited significantly attenuated systolic and diastolic blood pressure (Figures 5A–D). Furthermore, we assessed the impact on retinal microvascular using IB4 staining. This analysis revealed that inhibiting BACH1 expression successfully ameliorated the retinal vascular rarefaction characteristic of the hypertensive state (Figure 5E,F), indicating a restoration of angiogenic homeostasis.

To confirm the underlying molecular mechanism, we isolated primary aortic endothelial cells (MAECs) from these mice for further analysis. RT-qPCR results first validated the high efficiency of AAV9-mediated BACH1 knockdown in the target cells. Critically, in endothelial cells from the EC-KD group, the mRNA levels of FGF1, VEGFA, ANGPT1 and AGGF1 were significantly restored compared to the suppressed levels in the hypertensive group (Figure 5G). These findings were further corroborated at the protein level, as Western blot analysis demonstrated a parallel recovery in the expression of these key pro-angiogenic factors (Figures 5H,I).

Collectively, these *in vivo* data provided compelling evidence that endothelial BACH1 is a critical driver of both elevated blood pressure and impaired angiogenesis in hypertension, acting by suppressing a core set of pro-angiogenic factors.

**Figure 6:**
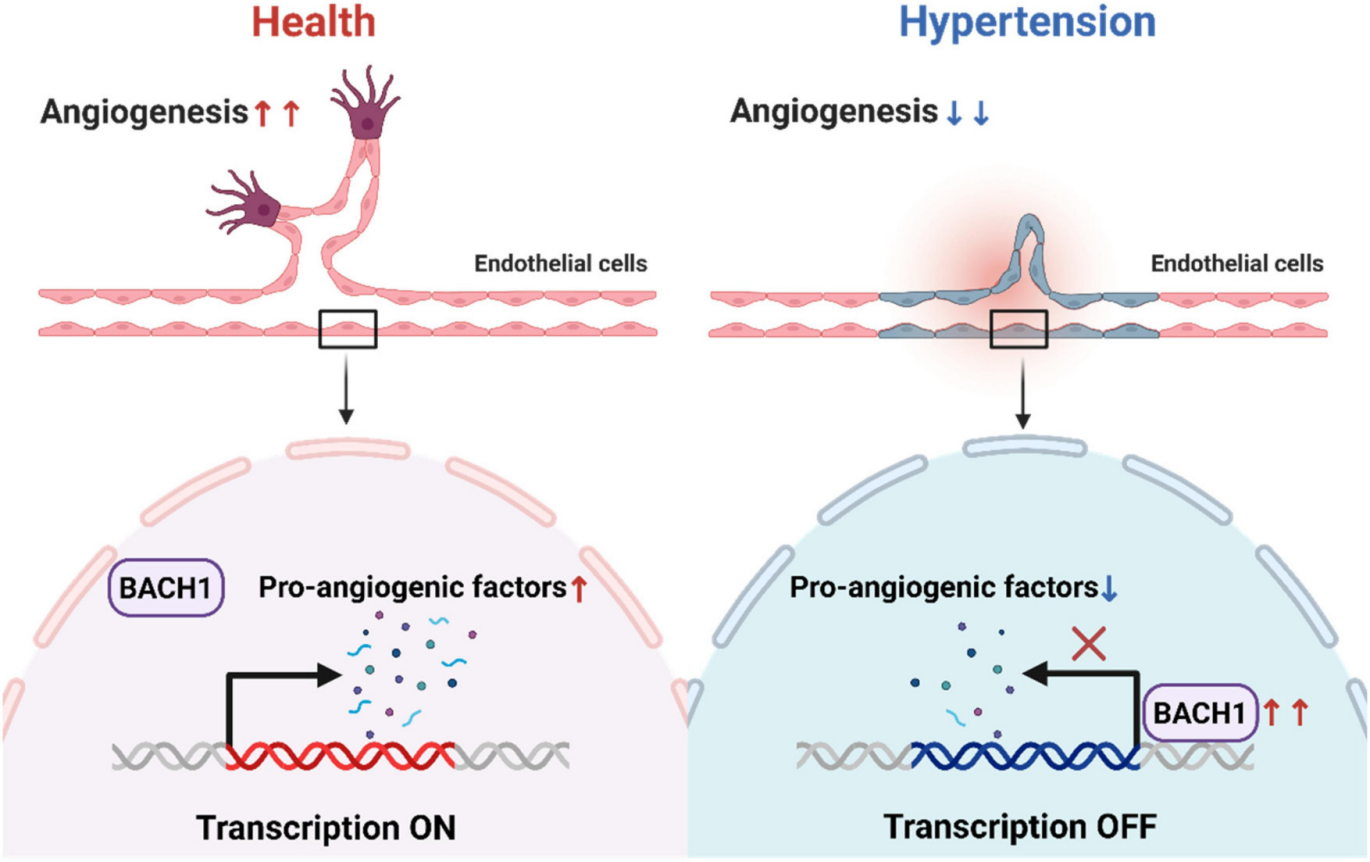
Schematic illustration. A schematic illustration showing that BACH1 caused angiogenic impairment in hypertension and showed its potential mechanisms. The illustration figure was created by BioRender.com.

In summary, our study identifies the transcription factor BACH1 as a pivotal molecule in the pathogenesis of hypertension, demonstrating its close association with both elevated blood pressure and impaired angiogenesis. Mechanistically, we reveal that under hypertensive conditions, BACH1 functions as a direct transcriptional repressor of a cohort of key pro-angiogenic factors, including FGF1, VEGFA, ANGPT1 and AGGF1. The coordinate suppression of these factors culminates in the deficient angiogenic response in hypertension (Figure 6). This work not only provides new insights into the molecular basis of hypertensive vascular disorders but also highlights that targeting BACH1 is a promising therapeutic strategy for improving angiogenesis and treating hypertension.

## 4 Discussion

A critical pathophysiological feature of hypertension is impaired angiogenesis (12, 19). However, the precise molecular drivers of this process remain to be fully investigated. In this study, we identify the transcription factor BACH1 as a previously unrecognized, pivotal mediator of angiogenic deficiency in hypertension. We provide a comprehensive line of evidence, from molecular mechanisms and clinical correlations to *in vivo* validation, demonstrating that BACH1 is upregulated under hypertensive conditions and acts as a direct transcriptional repressor of a core set of pro-angiogenic factors, thereby driving the impairment of angiogenesis.

The role of BACH1 as a transcriptional repressor is established previously, particularly in the context of cellular stress and heme metabolism (20, 21). BACH1 has also been increasingly implicated in inflammation and oxidative stress, which are also hallmarks of hypertension (15, 20, 22, 23). For instance, studies in atherosclerosis have shown that BACH1 deletion is protective (17). Emerging work suggests BACH1 promotes endothelial senescence (24). However, a functional role for BACH1 in regulating angiogenesis, particularly in the context of hypertension, had not been established. Our study provides the first evidence that AngII, a hypertensive mediator, serves as an inducer of endothelial BACH1 expression. This finding positions BACH1 as a critical molecule, driving the progression of hypertension.

Angiogenesis is regulated by a complex network of signaling molecules (25). While VEGFA is considered a master regulator, the process of angiogenesis requires a coordinated interplay with other factor families, such as angiopoietins, fibroblast growth factors, Notch signaling and AGGF1 (7, 8, 10, 11). Our work uncovers BACH1 as a common, direct repressor of a functionally diverse cohort of pro-angiogenic genes: *FGF1*, *VEGFA*, *ANGPT1*, and *AGGF1*. The coordinated suppression of FGF1, VEGFA, ANGPT1 and AGGF1 provides a mechanistic basis for the angiogenic impairment seen in hypertension.

The correlation between angiogenic factors and hypertension has been established before. For example, study has reported an inverse relationship between VEGF levels and the prevalence of hypertension (26). Our clinical data strongly reinforce and extend this observation. We not only confirmed significantly lower circulating levels of FGF1, VEGFA, and AGGF1 in hypertensive patients but also established a significant negative correlation between all four factors and both systolic and diastolic blood pressure. This strengthens the connection between our preclinical findings and pathophysiology of hypertension.

The most definitive evidence for a causal role of BACH1 is provided by our *in vivo* experiments. The fact that endothelium-specific BACH1 knockdown not only restored retinal vascular density but also significantly attenuated blood pressure is a crucial finding. This aligns with the concept that microvascular health is integral to blood pressure homeostasis (27). With small-molecule inhibitors of BACH1 currently under development for other diseases, such as cancer and pulmonary fibrosis (28, 29), our work provides a strong rationale for exploring BACH1 inhibition as a novel therapeutic strategy for hypertension.

## 5 Conclusion

In summary, this study delineates a novel and important pathway where BACH1 acts as the key regulator between hypertension and angiogenic impairment. By demonstrating that BACH1 suppresses a critical pro-angiogenic program, our work provides a new framework for understanding the vascular pathology of hypertension and validates BACH1 as a promising therapeutic target for hypertensive treatment.

## Data Availability

The original contributions presented in the study are included in the article/Supplementary Material, further inquiries can be directed to the corresponding author.
