# Supplementary material for: BACH1-mediated transcriptional repression of pro-angiogenic factors drives angiogenic impairment in hypertension
Source: Front Cardiovasc Med. 2026 Feb 11;13:1769747. doi: 10.3389/fcvm.2026.1769747 (PMC12932592; doi:10.3389/fcvm.2026.1769747)
Supplement: Supplementary file 1 [file Datasheet1.docx]

Supplemental Material for

BACH1-mediated transcriptional repression of pro-angiogenic factors drives angiogenic impairment in hypertension

Datian Gao^1^, Zhiwen Wu^1^, Zhiyin Zhou^1^, Jianwen Liang^1, *^

Correspondence to: [liangjw39@mail.sysu.edu.cn](mailto:liangjw39@mail.sysu.edu.cn)

**This file includes:**

Supplemental Figure 1-3

Tables S1 to S5

| 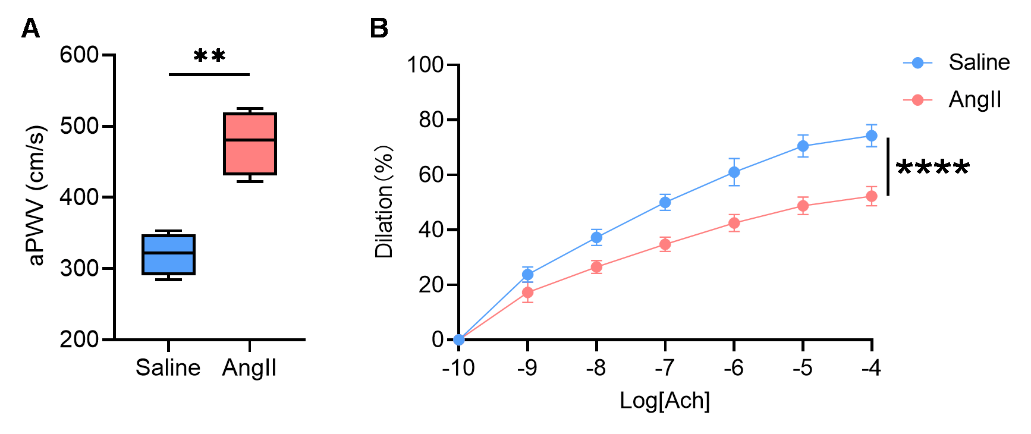 |
| --- |
| **Fig. S1: Hypertensive mice showed vascular endothelial injury.**  (**A**) *In vivo* aortic pulse wave velocity was measured by Doppler ultrasonography (n = 4). (**B**) The aortic ring assay was performed to evaluate the Ach-induced vasodilation (n = 4), The differences were analyzed between Saline and AngII group. Data were shown as mean ± s.d. ***P* < 0.01, *****P* < 0.0001. Statistical analysis was performed by two-tailed Student’s t-test (**A** and **B**). |

| 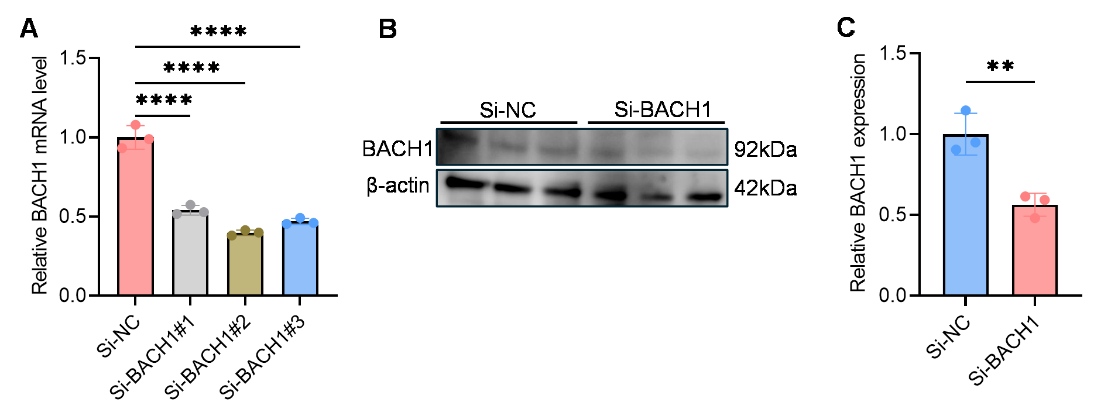 |
| --- |
| **Fig. S2: Validation of BACH1 Knockdown Efficiency.**  (**A-C**) mRNA (**A**, n = 3) and protein levels (**B** and **C**, n=3) validation of BACH1 knockdown efficiency in HUVECs via siRNA-BACH1 and negative control (NC). Data were shown as mean ± s.d. ***P* < 0.01, *****P* < 0.0001. Statistical analysis was performed by two-tailed Student’s t-test (C), by one-way ANOVA with Tukey’s post hoc test (**A**). |

| 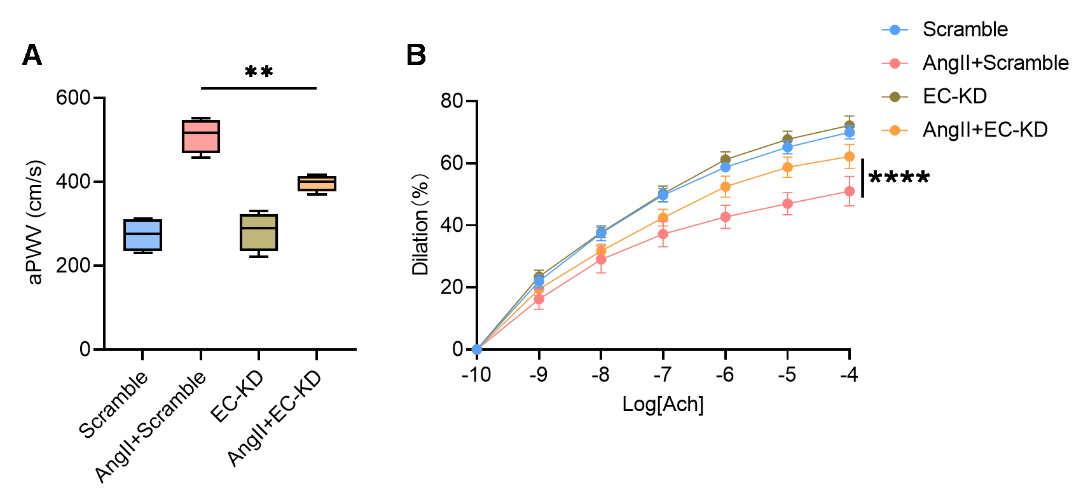 |
| --- |
| **Fig. S3: Endothelial-specific BACH1 knockdown improved endothelial function.**  (**A**) *In vivo* aortic pulse wave velocity was measured by Doppler ultrasonography (n = 4). (**B**) The aortic ring assay was performed to evaluate the Ach-induced vasodilation (n = 4), The differences were analyzed between AngII + Scramble and AngII + EC-KD group. Data were shown as mean ± s.d. ***P* < 0.01, *****P* < 0.0001. Statistical analysis was performed by one-way ANOVA with Tukey’s post hoc test (**A** and **B**). |

| **Supplementary Table 1. Clinical characteristics of hypertensive patients and healthy individuals** | | | |
| --- | --- | --- | --- |
|  | Health (n=30) | Hypertension (n=32) | P value |
| Demographics | | | |
| Age (years) | 49.27±6.38 | 49.53±6.31 | 0.87 |
| Male Sex [n (%)] | 16 (53.3) | 18 (56.3) | — |
| BMI (kg/m^2^)  Smoking [n (%)] | 19.94±2.11  5 (16.7) | 20.24±3.40  7 (21.9) | 0.68  — |
| BP |  |  |  |
| DBP (mmHg) | 74.27±7.11 | 92.47±7.90 | ＜0.0001 |
| SBP (mmHg) | 108.8±6.51 | 140.2±15.59 | ＜0.0001 |
| Metabolic indices |  |  |  |
| Fasting Serum Glucose (mmol/L) | 4.48±0.73 | 4.59±0.64 | 0.53 |
| Total Cholesterol (mmol/L) | 4.60±0.66 | 4.73±0.82 | 0.36 |
| LDL-C (mmol/L) | 2.67±0.71 | 2.95±0.82 | 0.16 |
| HDL-C (mmol/L) | 1.20±0.35 | 1.19±0.34 | 0.91 |
| Triglycerides (mmol/L) | 1.24±0.43 | 1.34±0.39 | 0.37 |
| Creatinine (μmol/L) | 66.82±11.90 | 65.36±13.56 | 0.66 |
| Uric Acid (μmol/L) | 358.8±49.5 | 364.7±58.1 | 0.67 |
| ALT (U/L) | 20.89±7.10 | 22.98±7.05 | 0.25 |
| AST (U/L) | 25.35±3.85 | 24.72±6.22 | 0.63 |

Supplementary Table 2. Sequences of Si-RNA

| **Gene** | **Sense (5’-3’)** | **Antisense (5’-3’)** |
| --- | --- | --- |
| Si-BACH#1 | GGAGAAGAAUGCUGCUCAATT | UUGAGCAGCAUUCUUCUCCTT |
| Si-BACH#2 | CCACCAGGUUUGAUGAUAUTT | AUAUCAUCAAACCUGGUGGTT |
| Si-BACH#3 | GGAGCUACUUCAAGAACAATT | UUGUUCUUGAAGUAGCUCCTT |

Supplementary Table 3. Primers for RT-PCR

| **Gene** | **Forward** | **Reverse** |
| --- | --- | --- |
| BACH1 | AGCCAACCTGACCCTGAAGA | GGTGGTTTCCATGCTGTCCT |
| BACH2 | CAGCCTGGAGAAGGTGAACG | TGCTTCTGGTCTTGGCTTTG |
| FGF1 | TACCAAGAACGGCAGCTTCT | TCCAGGTCCTTGAGCAGGTA |
| FGF2 | AGCGGCTCTACTGCAAGAAC | TCCGTTTTAGGCCACACACTC |
| AGGF1 | GACTGGGACTGGGAGAAGGT | GCTCCAGGTAGTGGCTTTGG |
| VEGFA | TGCTGCTCTACCTCCACCAT | CACAAGGGGCACAGGGATTT |
| VEGFB | GACCAAGAAACCGGAAGCAA | TCCTTGTACACACCTCCCTGA |
| ANGPT1 | CCAGCAGATGCAGAAACCAG | TGGTACTTGGGGTCGTTGTG |
| ANGPT2 | CCAGCCAACATCCAATCCTA | GGTTGGGCTCATACTTCTCCA |
| PIGF | GGCTGCTGGCTTTACTCATC | TGGCTTTGGTCTTGCTGGTA |
| PDGFA | GATGCCTGGAGAGTTGGAGT | TGGCACTTGTCGGTCTTGTA |
| NOTCH1 | ACCGAGTGTGATGGCTGAAT | TTGTTTCGCCACTGTCTTCA |
| ACTB | CCTGGCACCCAGCACAAT | GCCGATCCACACGGAGTACT |
| Bach1 (*Mus musculus*) | TGAAGCTGAGCAAGCTCAAC | GTCCTTGCTGTCCCAGTTCT |
| Fgf1 (*Mus musculus*) | GAGGCTGAAGAGCGAGAAGG | TCCTTGAGCAGGTAGTCGGT |
| Vegfa (*Mus musculus*) | GCACATAGAGAGAATGAGCTTCC | CTCCGCTCTGAACAAGGCT |
| Angpt1 (*Mus musculus*) | CCTGCAGAACCAGAACCAAG | TGGTACTTGGGGTCGTTGTG |
| Aggf1 (*Mus musculus*) | CAGCCAGGACCTCATTCAAC | GGAGGTAGTGGCTTTGGGTC |
| Actb (*Mus musculus*) | AGGCCCAGCTCAGTACCA | GGGGTGTTGAAGGTCTCAAA |

Supplementary Table 4. Antibodies used in western blot and immunofluorescence

| **Antibody** | **Supplier** | **Identifier** |
| --- | --- | --- |
| Rabbit Anti-Human BACH1 | Cell Signaling Technology | Cat# 33059 |
| Rabbit Anti-Human/Mouse BACH1 | Proteintech | Cat# 14018-1 |
| Rabbit Anti-Human BACH2 | Proteintech | Cat# 27635-1 |
| Rabbit Anti-Human/Mouse FGF1 | Proteintech | Cat# 17400-1 |
| Rabbit Anti-Human/Mouse VEGFA | Proteintech | Cat# 81323-2 |
| Rabbit Anti-Human ANGPT1 | Proteintech | Cat# 27093-1 |
| Rabbit Anti-Mouse ANGPT1 | Abcam | Cat# ab183701 |
| Rabbit Anti-Human/Mouse AGGF1 | Proteintech | Cat# 11889-1 |
| Rabbit Anti-Mouse AGGF1 | Abcam | Cat# ab203680 |
| Mouse Anti-Human/Mouse β-actin | Proteintech | Cat# 66009-1 |
| Anti-Rabbit IgG, HRP-linked antibody | Cell Signaling Technology | Cat# 7074 |

Supplementary Table 5. Primers for CHIP-qPCR

| **Gene** | **Forward** | **Reverse** |
| --- | --- | --- |
| FGF1 | GGCTGGGACTGGTTAAAGGA | CCCAAAGCAACCTCAACCAT |
| VEGFA | CCTCCTCTTCCTCTTCCTCC | GGCTTTCTTGCTCTTCCTTG |
| ANGPT1 | AGGAAGGGGACAGGAGAAGG | TCCCTTCCCTTCCTCTTCTC |
| AGGF1 | GGGAGGAAGAGGAAGGAGGT | CCTCCAACCTCCACCTCTTT |
