# Supplementary figures and images for: BACH1-mediated transcriptional repression of pro-angiogenic factors drives angiogenic impairment in hypertension
Source: Front Cardiovasc Med. 2026 Feb 11;13:1769747. doi: 10.3389/fcvm.2026.1769747 (PMC12932592; doi:10.3389/fcvm.2026.1769747)

Fig. 2B:

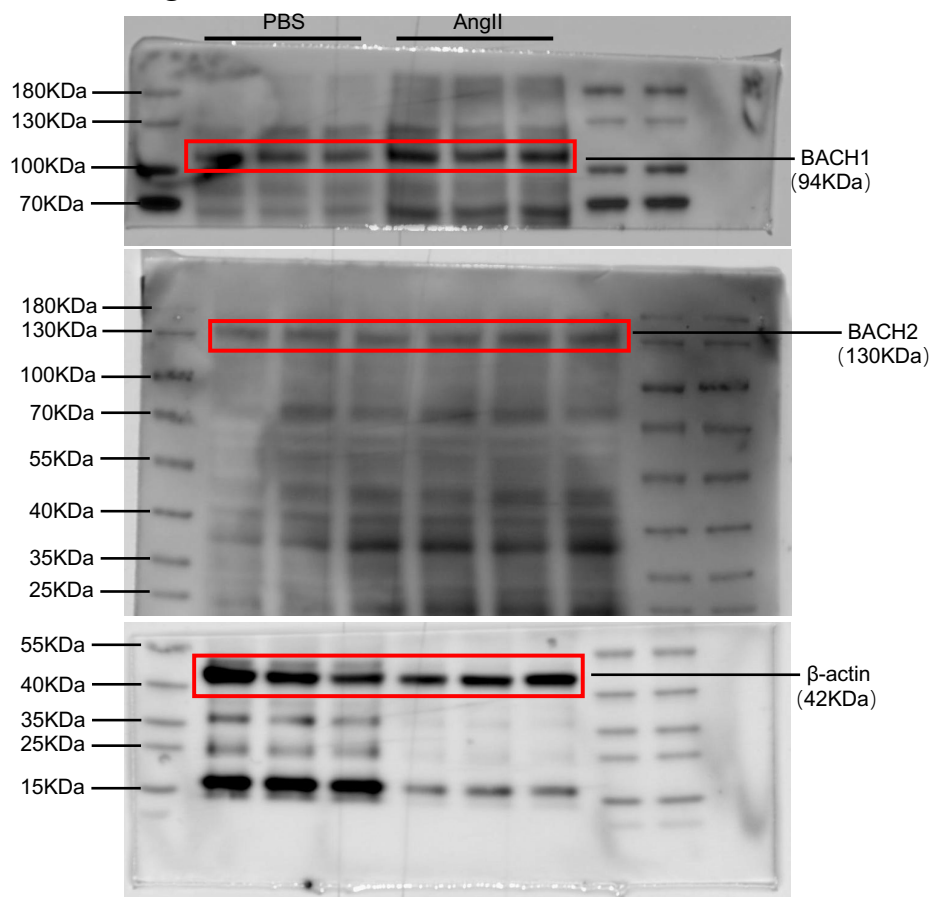

Fig. 3B:

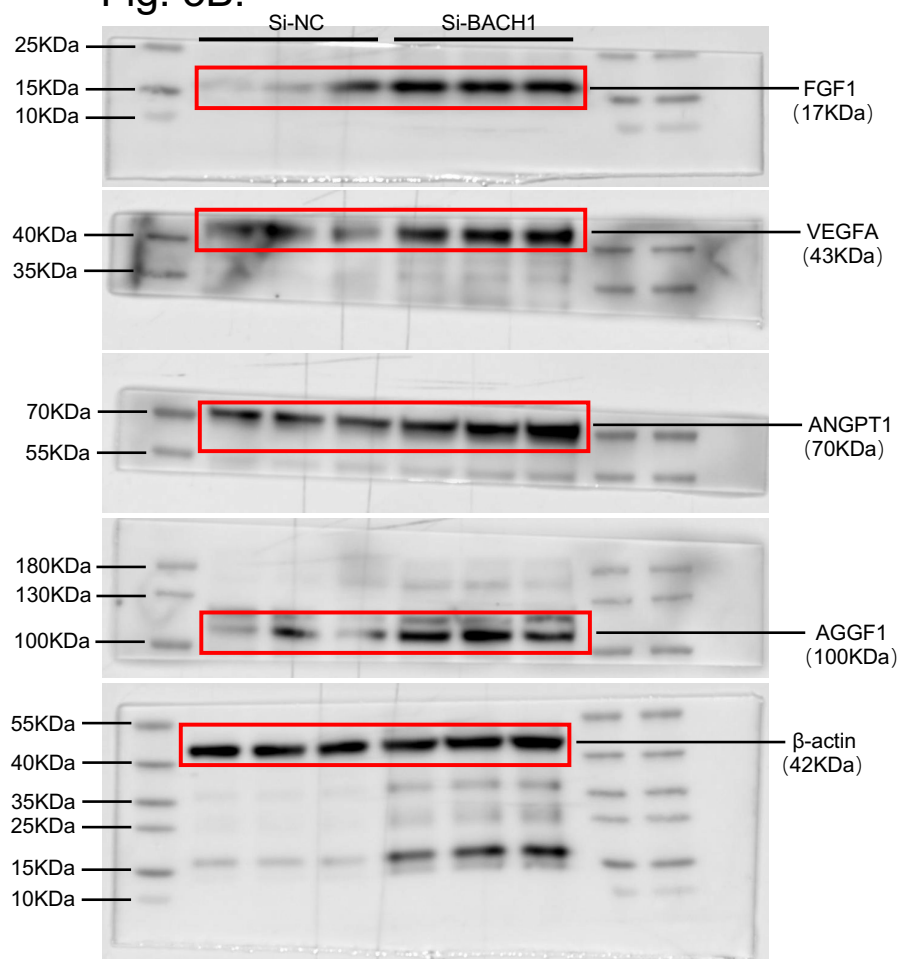

Fig. 3E:

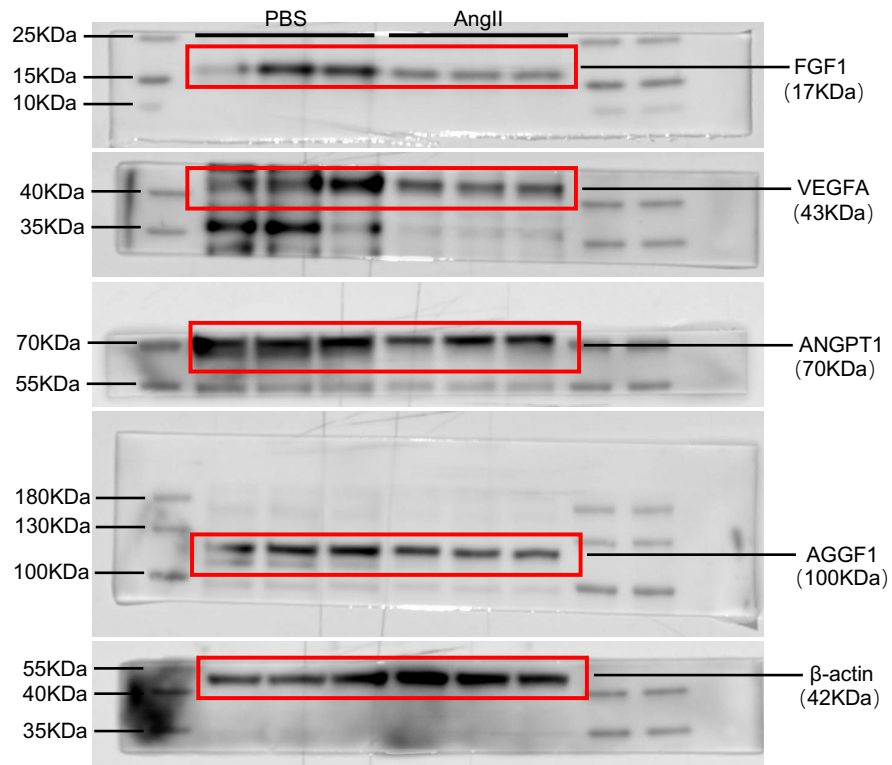

Fig. 3H:

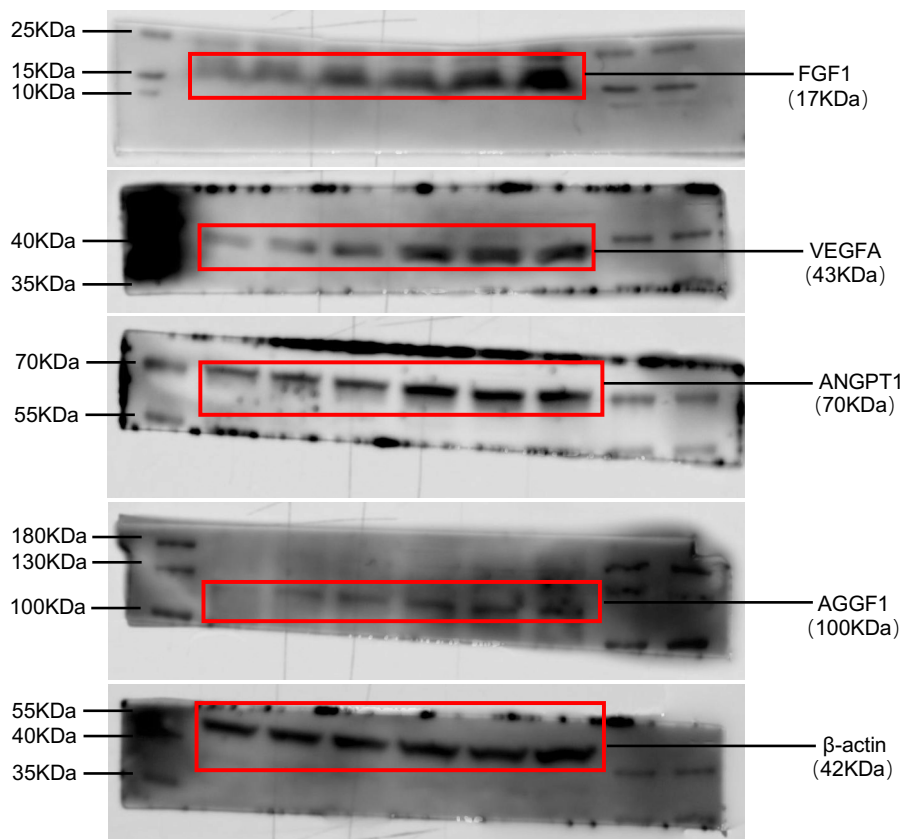

Fig. 5H:

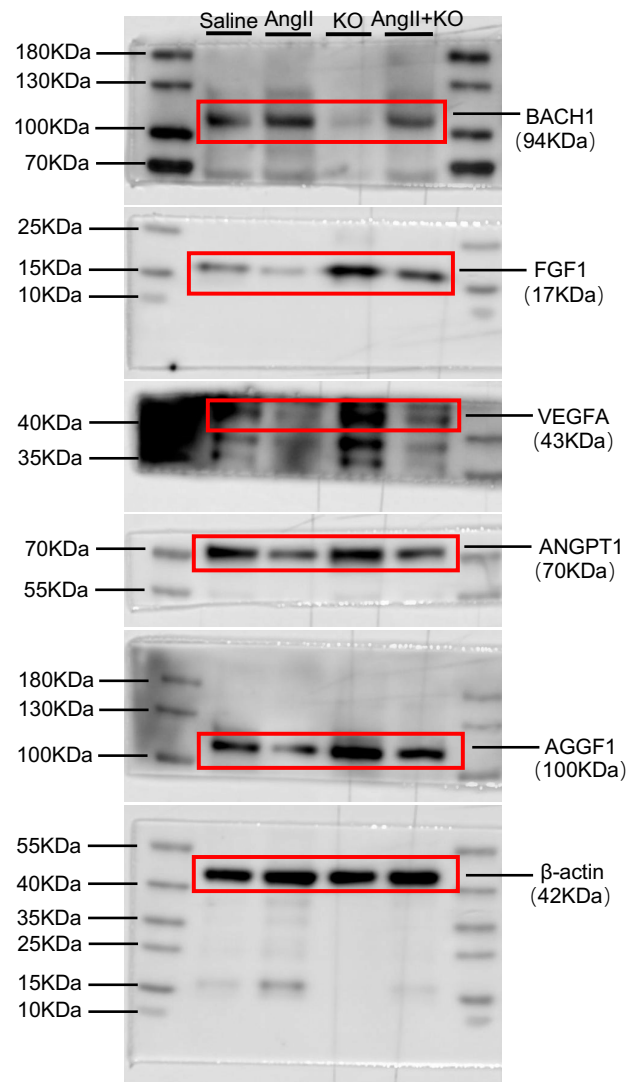

Fig. S2B:

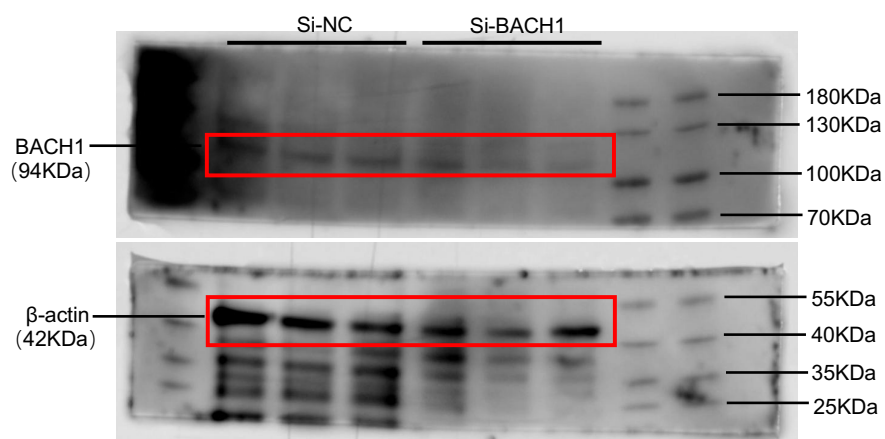

Supplement: Supplementary file 2 [file Datasheet2.pdf]
